# 14-day Holter monitoring for atrial fibrillation after ischemic stroke: The yield of guideline-recommended monitoring duration

**DOI:** 10.1177/23969873221146027

**Published:** 2022-12-23

**Authors:** Jelle CL Himmelreich, Wim AM Lucassen, Jonathan M Coutinho, Ralf E Harskamp, Joris R de Groot, Henk CPM van Weert

**Affiliations:** 1 Department of General Practice, Amsterdam UMC Location University of Amsterdam, Amsterdam, The Netherlands; 2 Amsterdam Public Health, Personalized Medicine, Amsterdam, The Netherlands; 3 Department of Neurology, Amsterdam UMC Location University of Amsterdam, Amsterdam, The Netherlands; 4 Amsterdam Neuroscience, Neurovascular Disorders, Amsterdam, The Netherlands; 5 Department of Cardiology, Amsterdam UMC Location University of Amsterdam, Amsterdam, The Netherlands; 6 Amsterdam Cardiovascular Sciences, Heart Failure & Arrhythmias, Amsterdam, The Netherlands

**Keywords:** Ischemic stroke, TIA, atrial fibrillation, screening

## Abstract

**Introduction:**

Current European Stroke Organisation (ESO) guidelines recommend >48 h of continuous electrocardiographic monitoring for atrial fibrillation (AF) in all patients with ischemic stroke or transient ischemic attack (TIA) with undetermined origin. We assessed the yield of the guideline-recommended monitoring for AF, as well as of extending monitoring up to 14 days.

**Patients and methods:**

We included consecutive patients with stroke/TIA without AF in an academic hospital in The Netherlands. We reported AF incidence and number needed to screen (NNS) in the overall sample after 48 h and 14 days of Holter monitoring.

**Results:**

Among 379 patients with median age 63 years (IQR 55–73), 58% male, Holter monitoring detected 10 cases of incident AF during a median of 13 (IQR 12–14) days of monitoring. Seven AF cases were detected within the first 48 hours (incidence 1.85%, 95% CI 0.74–3.81; NNS 54), and three additional AF cases were recorded among the 362 patients with >48 h of monitoring and without AF ⩽ 48 h (incidence 0.83%, 95% CI: 0.17–2.42; NNS 121). All AF cases were detected within the first 7 days of monitoring. Our sample was subject to sampling bias favoring inclusion of participants with low AF risk.

**Discussion:**

Strengths of this work were the broad inclusion criteria as recommended by ESO guidelines, and high Holter adherence among participants. The analysis was limited by inclusion of lower-risk cases and a relatively small sample size.

**Conclusion:**

In low-risk patients with recent stroke or TIA, ESO guideline-recommended screening for AF resulted in a low AF yield, with limited additional value of monitoring up to 14 days. Our results underline the need for a personalized approach in determining a patient’s optimum duration for post-stroke non-invasive ambulatory monitoring.

## Graphical abstract

**Graphical abstract:**
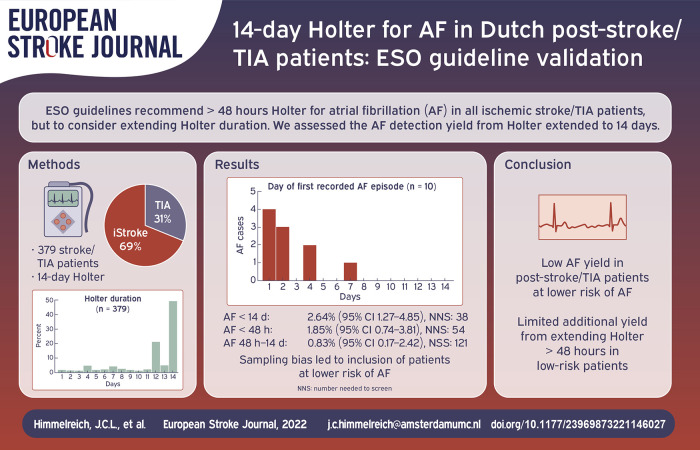

## Introduction

Atrial fibrillation (AF) is an arrhythmia associated with an elevated risk of ischemic stroke that can be effectively lowered with oral anticoagulation.^1^ Confirming AF on electrocardiogram (ECG) is however complicated due to its often paroxysmal and/or asymptomatic nature. Screening for paroxysmal AF is therefore warranted in high-risk populations in order to achieve early AF detection to lower the burden of (recurrent) stroke.^1,2^

It is estimated that up to a quarter of ischemic strokes are AF-related.^3,4^ Ambulatory post-stroke monitoring yields between 10.7% and 14.7% AF cases, with higher yield among selected patients.^5,6^ Therefore, in patients with recent brain ischemia there is consensus to screen for AF if no other cause for stroke is detected.^1,2,7,8^ However, there is no uniform recommendation on optimal rhythm monitoring duration.^2^ The European Stroke Organisation (ESO), in its Guideline on screening for subclinical atrial fibrillation after stroke or transient ischemic attack of undetermined origin (2022) recommends more than 48 h of cardiac monitoring for AF after stroke or transient attack (TIA). While the American Heart Association/American Stroke Association (AHA/ASS) provide no specific recommendations on monitoring duration,^8^ the European Society of Cardiology (ESC) recommends at least 72 h of post-stroke monitoring for AF in all patients with recent ischemic stroke or TIA.^1^ Guidelines generally advise to consider extending monitoring duration in order to increase the chance of detecting silent AF, without specific recommendations whom to select for such prolonged monitoring.^1,2,8–10^

We investigated the merits of guideline recommendations for post-stroke ECG monitoring duration by presenting the yield of post-stroke monitoring with 14-day ambulatory Holter in consecutive patients who presented with ischemic stroke or TIA and who were free of AF at inclusion. The primary aim of the current analysis was to assess the overall yield of newly diagnosed AF in an unselected post-stroke or TIA population using 14-day Holter. Secondary aims were to assess AF yields when monitoring according to, as well as beyond, the ESO and ESC guideline-recommended 48 and 72 h, respectively, and to assess AF yield in clinically relevant subgroups of ischemic stroke and TIA patients.

## Patients and methods

The current work is presented within the framework of the Risk Assessment for the Identification of Paroxysmal AF (RAPID-AF) study which was initiated to validate novel techniques of assessing the risk of new AF in a combined dataset two high-risk populations: post-stroke and elderly primary care patients [Netherlands Trial Register, NTR6489]. The current analysis aims to provide detailed results on AF yield in RAPID-AF’s post-stroke arm. Data on the primary care arm of the RAPID-AF study has been published previously.^11^

### Patients

For the post-stroke arm of the RAPID-AF study we included consecutive patients with ischemic stroke or TIA (stroke/TIA) without a history of AF and free of AF on admission ECG who presented to the Neurology department of the Amsterdam University Medical Centers, location Academic Medical Center (AUMC-AMC). We defined ischemic stroke and TIA as an acute loss of focal cerebral or ocular function with symptoms lasting more than or under 24 hours, respectively, and which after adequate investigation was presumed to be due to embolic or thrombotic vascular disease.^7^ Inclusion was active from 18 July 2017 through 12 March 2020, and from 11 June 2020 through 17 December 2020, with the intermission and premature end date (distributing *n* = 400 out of the protocol’s stated aim of *n* = 500 Holter monitors) determined by clinical research restrictions in AUMC-AMC relating to the SARS-CoV-2 pandemic.

Patients were eligible for inclusion in the post-stroke RAPID-AF ambulatory Holter monitoring cohort if they: neither had a history of AF nor de novo AF on ambulance, admission or inpatient ECG or bedside monitor before Holter initiation; were 18 years or older; did not use oral anticoagulation; were free of a pacemaker and/or implantable cardioverter defibrillator; had a life expectancy ⩾1 year as estimated by the neurologist in charge; would be able to wear a Holter device for 14 days, and; provided informed consent. We excluded patients who had an alternative explanation for stroke or in whom AF-related stroke was highly unlikely (e.g. periprocedural stroke, symptomatic internal carotid artery (ICA) dissection, or symptomatic ICA occlusion). In patients who presented more than once to our clinic during active inclusion we assessed eligibility only at the first encounter for stroke/TIA.

### Study procedures

Standard of care in our center for those presenting with stroke/TIA at time of enrollment consisted of clinical assessment, brain imaging (CT in all patients; MRI at the discretion of the physician e.g. in case of doubt regarding diagnosis or stroke location with consequences for treatment), 12-lead resting ECG, laboratory tests, carotid imaging by ultrasound and/or CT-angiogram in case of non-vertebrobasilar stroke/TIA, and ambulatory Holter monitoring (up to 14 days when consenting to RAPID-AF study participation, or up to 72 h in absence of study consent). Initiation and duration of bedside cardiac monitoring in those admitted for stroke was at the discretion of the physician. Patients with TIA or mild stroke generally were not admitted. Patients aged 50 or younger at presentation were given more elaborate investigation including additional laboratory tests, brain MRI and echocardiography as part of the young stroke protocol. For other patients, such additional investigations were at the discretion of the treating physician. Study Holter was the first form of ambulatory cardiac monitoring in all patients included in RAPID-AF.

Additional study procedures were as follows. A study nurse included eligible patients either in the Neurology department, the emergency unit or in the RAPID-AF study outpatient clinic. We aimed to include patients within 90 days after onset of symptoms of the qualifying stroke/TIA. After informed consent, the nurse collected baseline data and instructed patients on the use of the Holter device (Fysiologic ECG Services, Amsterdam, The Netherlands). The study used 2-lead Holters corresponding to leads V1 and V5 of the standard 12-lead ECG, with 8 bit resolution and sampling rate 100 Hz. The leads were applied on the patient’s body by three patches attached to one wire leading to a wallet-sized device which was worn in a pouch around the patient’s neck. We instructed patients to wear the device continuously except when bathing. We encouraged patients to wear the Holter for the maximum of 14 days, but indicated that they were free to return the device earlier. We instructed patients to return their device either at a return clinical visit or through a prepaid return envelope provided by the study team.

Study procedures were in accordance with the Declaration of Helsinki on medical research involving human subjects.

### Baseline data

We collected baseline data at the baseline visit and from the hospital’s electronic health records (EHR). At the baseline visit, study personnel asked the patient for data on ethnicity, family history for AF, height, weight, smoking and alcohol consumption. Baseline data retrieved from the EHR consisted of age, sex, index ischemia type and location, stroke severity as per the National Institutes of Health Stroke Scale (NIHSS; score ranging from 0 to 42 with higher scores indicating clinically more severe stroke),^12^ blood pressure, baseline 12-lead ECG parameters, medication use, medical history, and relevant routine care laboratory findings. We defined stroke/TIA location as either retinal, vertebrobasilar, lacunar or non-lacunar anterior, middle or posterior cerebral artery (ACA, MCA, and PCA, respectively) territory, as assessed by clinical symptoms (primarily) and/or available brain imaging. We distinguished the subgroup of patients with non-lacunar hemispheric stroke, defined as retinal, ACA, MCA, or PCA ischemic stroke, due to its relevance in post-stroke AF detection.^13,14^ We were unable to distinguish other subgroups relevant to post-stroke AF detection such as cryptogenic stroke or embolic stroke of undetermined source due to lack of systematic pre-enrollment cardiac monitoring and/or echocardiography in our center’s standard post-stroke/TIA work-up (see under “Study procedures”).^14,15^

We defined vascular disease as history of coronary artery disease, myocardial infarction, peripheral arterial disease, aorta dilatation or known arterial plaques. We defined prior cardiac intervention as a history of coronary artery bypass grafting, percutaneous coronary intervention or cardiothoracic surgery. We determined stroke location based on brain imaging and/or clinical symptoms. We calculated the CHA_2_DS_2_-VASc score from baseline medical history EHR data.^16^

In order to assess potential sampling bias we collected a selection of baseline variables from stroke/TIA presentations from a random sample of 25% of potentially eligible patients who were not included in our study, as permitted by the Dutch Medical Research Involving Human Subjects Act (WMO) on the use of de-identified routinely collected medical data. In those excluded for a de novo AF diagnosis, we recorded the time at which the AF diagnosis was made (at presentation for, during admission for, or after discharge for their stroke/TIA).

### Outcome definitions

The primary outcome was the overall incidence of newly diagnosed (incident) AF as per ambulatory Holter monitoring, with AF defined as AF or atrial flutter lasting ⩾30 s.^17^ Fysiologic ECG Services (Amsterdam, The Netherlands) performed analysis of all study Holters through digital pre-selection of relevant recordings, followed by manual assessment by trained cardiologists.

Secondary outcomes were the number of days until first AF detection in those with AF diagnosed on study Holter, and AF incidence during monitoring after the guideline-recommended 48 h of Holter monitoring in those who wore their Holter >48 h.^2^

### Statistical analysis

We reported medians and interquartile range (IQR) for continuous variables, and numbers and percentages for categorical variables. In case of missing data we reported the percentage of missing data for each baseline variable with missingness. We displayed baseline characteristics at first presentation for the overall sample, as well as stratified by AF presence on Holter. We plotted the distribution of first day of AF diagnosis in those with AF on Holter, as well as the distribution of the number of days of Holter recording per patient. We calculated incidence and 95% confidence interval (95% CI) and number needed to screen (NNS) using the exact method for AF diagnosed during overall (up to 14 days) Holter monitoring as well as at 24, 48, and 72 h in order to assess the merits of different guidelines.^1,2,10^

We provided a sensitivity analysis of AF incidence and factors associated with AF detection in those with Holter duration over 48 h and without AF detected in the first 48 h of monitoring, in order to assess the added value of monitoring beyond ESO’s currently recommended 48 h post-stroke.^2^

In order to assess whether the application of selection criteria for post-stroke monitoring could have increased AF yield, we presented AF incidence and NNS in subgroups of patients with stroke (not TIA), non-lacunar hemispheric stroke/TIA, and patients with moderate-severe stroke at presentation (NIHSS ⩾ 7),^6,18,19^ and we presented discrimination of multivariable prediction models developed for post-stroke AF detection. We assessed model discrimination by the C-statistic and 95% CI, using the AS5F (Age, Stroke Severity NIHSS > 5 to Find AF),^20^ Re-CHARGE-AF (Recalibrated Cohorts for Heart and Aging Research in Genomic Epidemiology for Atrial Fibrillation), and STAF (Score for the Targeting of AF)^21^ risk models, with risk calculated for each patient at baseline using the models’ originally published coefficients, and with 95% CIs calculated using 2000 bootstrap samples.

We used R version 3.6.1^22^ using the epiR, expss, ggplot2, lubridate, pROC, scales, and Table 1 packages for our analyses.

**Table 1.** Main baseline characteristics of the study sample.

	Overall (*n* = 379)
Female sex (n, %)	160 (42.2)
Age, years (Median [Q1–Q3])	63.0 (55.0–73.0)
Ethnicity	
Caucasian/white (n, %)	261 (68.9)
African/black (n, %)	71 (18.7)
South Asian (n, %)	19 (5.0)
Asian (n, %)	18 (4.7)
Other (n, %)	10 (2.6)
Qualifying event	
Ischemic stroke (n, %)	261 (68.9)
TIA (n, %)	118 (31.1)
Stroke/TIA location	
Middle cerebral artery (n, %)	169 (44.6)
Anterior cerebral artery (n, %)	7 (1.8)
Posterior cerebral artery (n, %)	20 (5.3)
Lacunar (n, %)	47 (12.4)
Retinal (n, %)	16 (4.2)
Vertebrobasilar (n, %)	120 (31.7)
Non-lacunar hemispheric stroke (n, %)	131 (34.6)
NIHSS at first presentation (Median [Q1-Q3])	1 (0–3)
Ipsilateral carotid artery stenosis >50% (n, %)	10 (2.6)
Intravenous thrombolysis (n, %)	74 (19.5)
Intra-arterial thrombectomy (n, %)	27 (7.1)
Time to Holter, days (Median [Q1–Q3])	35 (14–60)
Time to Holter ⩽90 days (n, %)	337 (88.9)
Holter duration, days (Median [Q1–Q3])	13 (12–14)
Heart failure (n, %)	10 (2.6)
Hypertension (n, %)	176 (46.4)
Diabetes (n, %)	71 (18.7)
Prior myocardial infarction (n, %)	29 (7.7)
Prior stroke/TIA/SE (n, %)	83 (21.9)
Vascular disease (n, %)	49 (12.9)
CHA_2_DS_2_-VASc (Median [Q1–Q3])	4 (3–5)
Antiplatelet use (n, %)	130 (34.3)
ACE/ARB use (n, %)	109 (28.8)
Calcium antagonist use (n, %)	70 (18.5)
Diuretics use (n, %)	50 (13.2)
Statin use (n, %)	140 (36.9)
Insulin use (n, %)	20 (5.3)
Metformin use (n, %)	49 (12.9)

ACE/ARB: angiotensin-converting enzyme inhibitor/angiotensin II receptor blocker; CHA2DS2-VASc: congestive heart failure, Hypertension, Age ≥75 years (doubled), Diabetes mellitus, prior Stroke or TIA or thromboembolism (doubled), Vascular disease, Age 65 to 74 years, Sex category; NIHSS: National Institutes of Health Stroke Scale; Q1: 1st quartile; Q3: 3rd quartile; SE: systemic embolism; TIA: transient ischemic attack.

## Results

**Figure 1.**
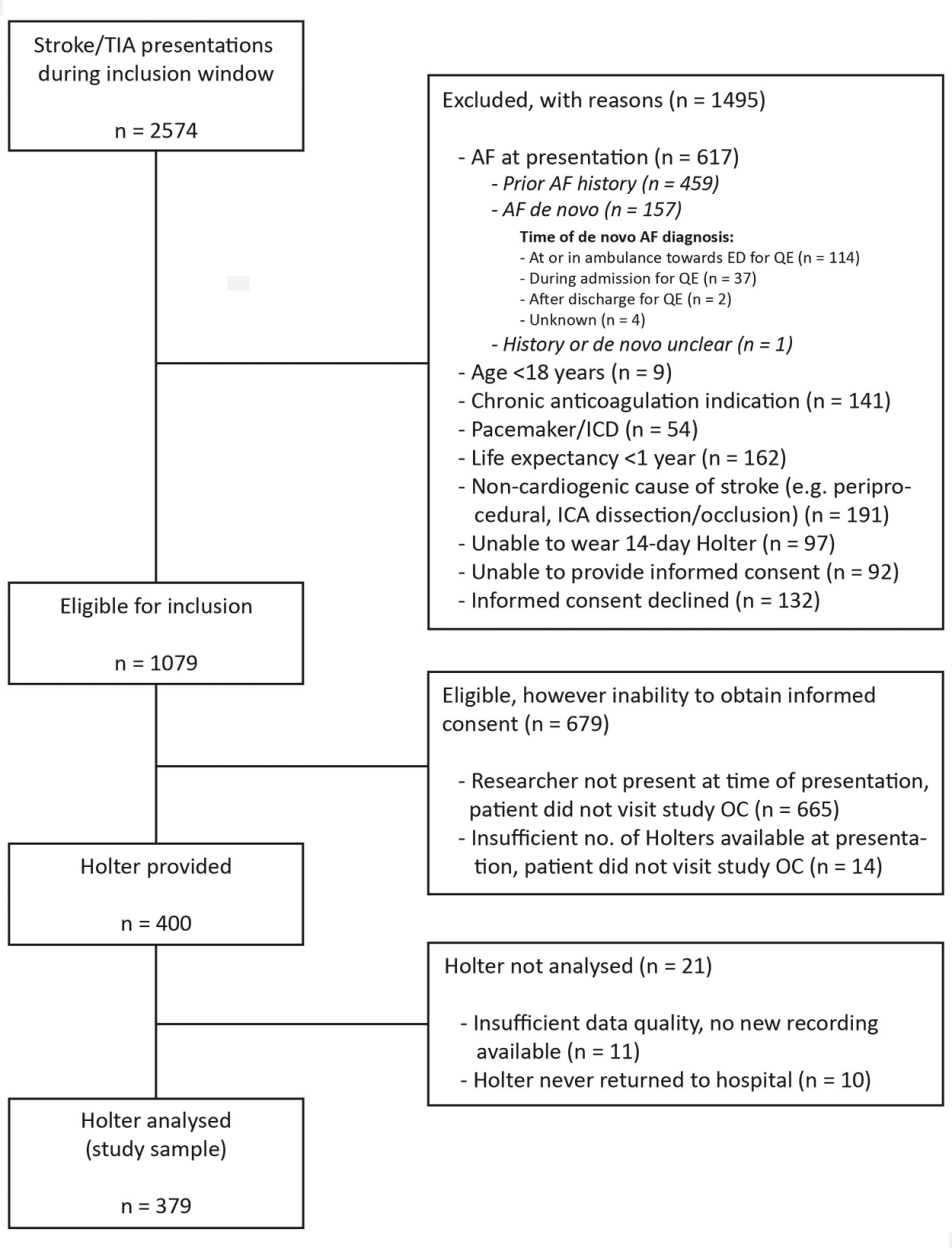
Study flowchart. AF: atrial fibrillation; ED: emergency department; ICA: internal carotid artery; ICD: implantable cardioverter defibrillator; OC: outpatient clinic; QE: qualifying event; TIA: transient ischemic attack.

Out of 2574 patients who presented with stroke/TIA during the study inclusion windows, 1079 patients (41.9%) were eligible for inclusion (see flowchart, Figure 1). Of these, 400 patients provided written consent and were given a study Holter. We collected analyzable Holter recordings from 379 of these 400 patients, constituting the study sample (35.1% of all eligible patients).

Of the 1495 patients with one or more exclusion criteria, the main reason for exclusion was known AF at time of study eligibility assessment (*n* = 617, 41.3%). Of these, 157 (25.4%) were de novo AF diagnoses, a majority of whom were diagnosed at first presentation (Figure 1).

### Patient characteristics

**Figure 2.**
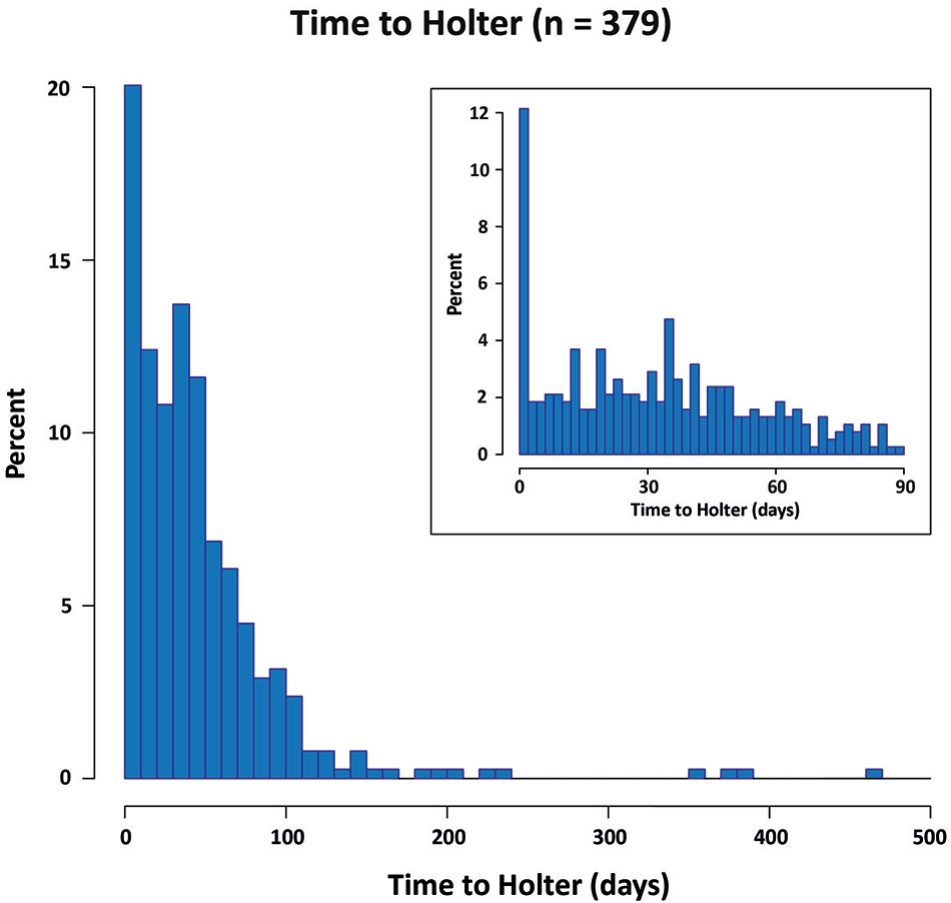
Time between qualifying event and Holter initiation (*n* = 379). Insert: detailed view of time between qualifying event and Holter initiation as percentage of total patients within the first 90 days after qualifying event.

Table 1 shows the main baseline characteristics of included patients. Median age of the included patients was 63 years (IQR: 55–73), 57.8% was male and 69% Caucasian/white. Most patients were included with stroke as qualifying event (68.9% vs 31.1% with TIA), with the middle cerebral artery (MCA) being the most common location for stroke/TIA. Median NIHSS of the total sample was 1 (IQR: 0–3). Median CHA_2_DS_2_-VASc was 4 (IQR: 3–5). Platelet inhibitors and statins were used at presentation by 34.3% and 36.9%, respectively. Time to Holter was median 35 days (IQR: 14–60), with 88.9% of Holters initiated within 90 days (Figure 2).

Supplemental Table S1 shows a comparison of baseline characteristics between the study sample and a 25% random sample of non-included eligible patients. Included patients were younger and more often female, had lower NIHSS at presentation, more often had a TIA as qualifying event, less often had an MCA stroke, less frequently underwent intravenous thrombolysis (IVT) or intra-arterial thrombectomy (IAT), and had lower cardiovascular comorbidity and medication use. Of those eligible but not included, 30.1% were presented to our center for tertiary IAT care after which they were discharged to their referring secondary care centers. A further 26.6% were discharged during admission due to shortage of beds in our center and/or residence outside the Amsterdam region.

### Holter results

**Figure 3.**
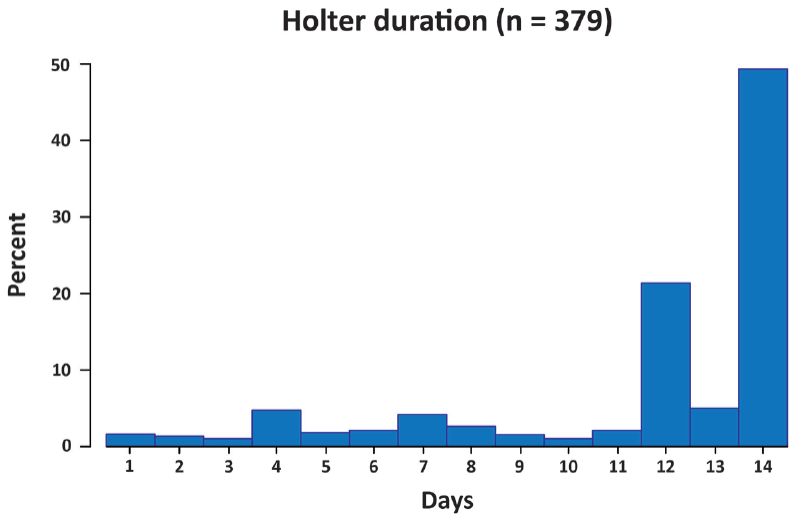
Total Holter recording duration in the overall sample (*n* = 379).

**Figure 4.**
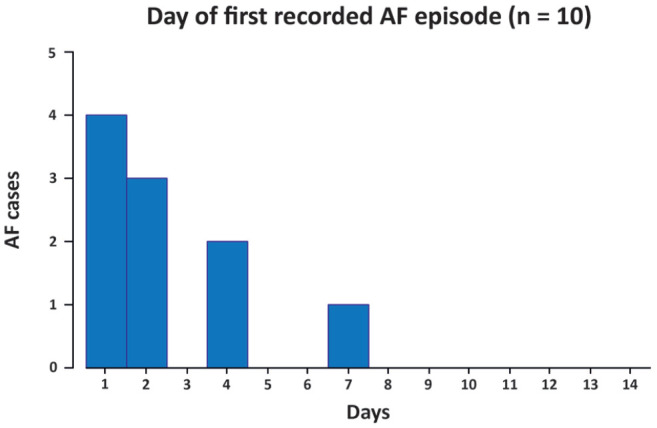
Day of first AF recorded in those with AF on Holter (*n* = 10). AF: atrial fibrillation.

Patients wore their Holter for a median of 13 (IQR: 12–14) days, with 96.0% wearing the device more than 3 days, and 83.1% using the Holter more than 7 days (Figure 3). Overall, 14-day study Holter recorded 10 AF diagnoses (2.64%, 95% CI 1.27–4.85; NNS 38). Four cases were diagnosed in the first 24 h of Holter monitoring (incidence 1.06%, 95% CI 0.29–2.70; NNS 94), and seven were recorded within the first 48 hours with no additional cases in the third day of monitoring (48- and 72-h incidence 1.85%, 95% CI 0.74–3.81; NNS 54). All cases in our sample were detected within the first week of Holter monitoring (Figure 4). Time to Holter initiation was not associated with AF detection in our sample (Supplemental Figure S1).

### Baseline characteristics and AF detection

There were a number of notable differences in baseline characteristics between patients who were and who were not diagnosed with AF on 14-day Holter (Supplemental Table S2). These included higher age (median 69.5 vs 63.0 years), higher rate of IAT (40.0% vs 6.2%) or IVT (50.0% vs 18.7%) on admission, higher rate of diuretics use (40.0% vs 12.5%), and lower serum triglycerides (median 0.90 vs 1.36 mmol/l) among those with and those without AF on 14-day monitor. Time from index stroke/TIA onset to Holter initiation was similar in both groups (median 45 days, IQR: 8–60, among those with and 35 days, IQR: 14–59, in those without AF).

### Patients with Holter duration >48 h

There were 362 patients who had worn their Holter >48 h and without documented AF during the first 2 days of monitoring (95.5% of the study sample). During the following median 12 days (IQR: 10–12), the study Holter detected three additional AF cases (0.83%, 95% CI 0.17–2.42; NNS 121). Baseline characteristics of patients with >48 h of Holter recording available are shown in Supplemental Table S3. Notable differences were seen in age (median 70.0 and 63.0 years), NIHSS (median 18 vs 1), proportion of patients who underwent IAT at presentation (66.7% vs 5.6%), and family history of AF (66.7% vs 12.4%) in those with and those without AF on Holter beyond the first 48 h, respectively.

### Subgroup analysis and prediction models

Among patients with ischemic stroke as qualifying event 9/261 had an AF during 14-day monitoring (incidence 3.45%, 95% CI 1.58–6.55; NNS 29). In patients with non-lacunar hemispheric stroke/TIA 9/212 had AF (incidence 4.25%, 95% CI 1.94–8.06; NNS 24), and among those with non-lacunar hemispheric stroke (not TIA) 8/131 showed AF on 14-day Holter (incidence 6.11%, 95% CI 2.64–12.03; NNS 16). Among patients with moderate-severe stroke at presentation 5/32 were diagnosed with AF during 14-day monitoring (incidence 15.63%, 95% CI 5.07–36.46; NNS 6).

C-statistics (95% CI) of the AS5F, Re-CHARGE-AF, and STAF risk models for 14-day AF detection were 0.75 (0.61–0.89), 0.59 (0.43–0.75), and 0.70 (0.56–0.84), respectively.

## Discussion

In low-risk patients with recent ischemic stroke or TIA attending an academic hospital in The Netherlands, screening for AF using 14-day Holter resulted in an overall AF yield of 2.6%, with an NNS of 38. Extending Holter monitoring beyond the ESO guideline-recommended 48 h for post-stroke patients resulted in 0.83% of new AF cases (NNS 121), with no new AF cases detected after the first 7 days of ambulatory monitoring within our study sample. Selection of higher-risk patients to screen for AF using clinically relevant criteria would have increased AF yield. Our results underline the need for a personalized approach in determining a patient’s optimum duration for post-stroke non-invasive ambulatory monitoring.

### Comparison with previous work

The incidence of device-detected AF during 14-day Holter monitoring in our sample was considerably lower than generally found in previous studies of prolonged ambulatory Holter monitoring among unselected patients with recent stroke/TIA.^5,6,23,24^ Even among patients further selected for cryptogenic stroke, thus having undergone at least 24 h of continuous ECG monitoring before further monitoring, AF yield was generally higher in the first 14 days of recording than in our sample.^25–29^ Studies that reported Holter-detected post-stroke AF yields similar to ours were generally performed with 24-h rather than 14-day Holter^30–32^ or involved retrospective rather than prospective data.^33^

Potentially the main reason for the low AF yield was sampling bias toward inclusion of patients with a lower overall likelihood of post-stroke AF detection. The logistics of our inclusion process, with the requirement of active consent before Holter installation and with inclusion in part through a study outpatient clinic, has likely contributed to the undersampling of patients who attended our hospital only for IAT (short admission time) or with severe stroke (barriers to attending the study outpatient clinic) who are known to have a higher likelihood of post-stroke AF detection.^34^ As shown in our comparison between included patients and the random sample of eligible non-included patients, this resulted in a sample of patients who were younger, with less severe strokes and lower overall cardiovascular risk factor burden than the overall stroke/TIA population. Since AF is associated with increased stroke severity,^35,36^ and studies have shown clinical stroke scores to be associated with post-stroke AF detection,^34^ inclusion of lower-NIHSS patients could have led to a lower proportion of patients with AF in our study than in the overall stroke/TIA population. Likewise, IAT, an intervention to remove large thrombi from the intracranial anterior circulation, is typically performed in patients with higher stroke severity.^37,38^ It can be theorized that such thrombi are more frequently associated with AF, whether directly through AF-associated cardiac emboli or as a result of a cardiovascular risk profile that increases the risk of both large thrombi and atrial cardiomyopathy, including AF.^39^ It is therefore likely that the AF yield in our sample cannot be extrapolated to reflect that of the general Dutch stroke/TIA population.

In previous AF screening studies of unselected post-stroke/TIA patients participants were generally older.^23,40–42^ With age being one of the most important risk factors for AF incidence,^1^ this is likely to explain to a large degree the higher AF incidence seen in these studies. The proportion of TIA versus stroke as qualifying event as well as overall NIHSS in our sample were similar compared to previous unselected TIA/stroke studies on post-stroke AF.^40–42^ As these studies did not provide a comparison with potentially eligible non-included patients it is not known to what extent their baseline characteristics were affected by sampling bias.

Another possible explanation for the low AF incidence is the time from the qualifying event to commencement of Holter monitoring. Although data suggesting this association is limited, time to Holter initiation is likely related to the probability of AF detection after stroke.^2,43–45^ Research on the Stroke-Heart Syndrome (SHS) has indicated that post-stroke major adverse cardiovascular events and AF peak in the first 3–30 days after stroke onset.^45^ Our data showed no significant difference in time to Holter between patients with and without detected AF as reported in previous work.^28,46^ However, with median time to Holter of 35 days in our sample, we largely included patients outside the peak window for potential SHS cases. Our time to monitoring initiation far exceeded that of other studies which often had timely monitoring commencement in their inclusion criteria.^23,40^ These and other post-stroke studies with shorter mean duration to Holter have shown higher rates of AF detection.^27,42,46^ Our study excluded a number of patients with AF detected during admission for their stroke/TIA as de novo AF cases. As such cases may have been included in previous studies on post-stroke Holter yield, which would hinder a comparison with our work, we explicitly reported time of de novo AF detection in our Flowchart for comparison purposes. It is not known to what extent a further reduction in the time to Holter would have resulted in a higher AF yield in our population, or whether the sampling bias alone sufficiently accounts for the lower AF yield in our sample. In line with work on the SHS and its peak in the first 30 days, one could even speculate that the higher AF rate in studies with monitoring initiation directly after stroke were higher due to transient stroke-induced cardiomyopathy which we “missed” with mean 35 days to Holter initiation.^45^ A post-hoc sensitivity analysis applying inclusion criteria of previous unselected stroke/TIA AF screening studies to our data showed no significant increase in AF yield compared to our overall sample (data not shown),^23,40^ however sample size and event rate were severely limited in these analyses as in the overall analysis. Thus, the question whether every patient with stroke or TIA would benefit from rhythm monitoring directly following their cerebral event – which is currently not routinely performed in our center for logistical reasons – remains unanswered from our data.

A final explanation for our low AF yield is the proportion of patients who already had a known AF diagnosis at time of assessment for study Holter eligibility – a quarter of whom had de novo AF in our study. With pre-Holter AF prevalence of 41% in our stroke population this was considerably higher than in previous post-stroke AF screening studies that reported prior AF as reason for exclusion (generally below 20%).^30,32,47–50^ This potentially indicates that, in the presence of an already high proportion of post-stroke patients with (a history of) documented AF, prolonged rhythm monitoring has a relatively low additional yield. This view is supported by a recently published randomized controlled AF opportunistic screening trial among Dutch primary care patients of 65 years and over. The intervention did not achieve higher AF yield than usual care over a 1-year period, mainly underscoring the efficacy of detecting AF in routine primary care in the Netherlands.^11,51^

Our work subscribed to previous work which indicated that stroke location among patients with AF on post-stroke monitoring was most often non-lacunar hemispheric, with none or very few AF cases among patients with lacunar stroke/TIA.^6,41^ We note here that stroke location in our data was primarily based on clinical symptoms as MRI was not routinely performed in all stroke/TIA patients. As in previous work, we saw most AF cases detected during the first days of monitoring.^52^ Our data also concur with a recent systematic review and meta-analysis which found an association between IVT treatment, higher age, and lower triglycerides with AF detection.^34^ The yield of post stroke rhythm monitoring may thus increase when clinical risk factors for AF are taken into account.

### Clinical implications

Our findings are relevant for neurologists in similar settings who aim to optimize efficacy of their post-stroke rhythm monitoring strategy in low-risk stroke/TIA patients. The current data show that the yield of 48-h ambulatory Holter monitoring in a low-risk post-stroke patients of a Dutch academic hospital was lower than expected based on recent international literature. The additional value of monitoring beyond 48 h or 72 h as recommended by the ESC and similarly by the Dutch Neurological Society, was even more limited.^1,53^ Given that we detected a minority of cases within the first 24 h, 24-h monitoring as currently recommended by The National Institute for Health and Care Excellence (NICE) may be too short for post-stroke AF diagnosis.^10^ It is not known to what extent the more recent NICE diagnostic guidance to consider implanting implantable cardiac monitors in cryptogenic stroke patients will contribute to the detection of occult AF after brain ischemia.^54^

Recent publications have emphasized that the optimal screening strategy for AF after stroke/TIA is yet to be determined.^55^ The current work underlines the potential for a more personalized approach than the current recommendation to screen all stroke/TIA patients for more than 48 h.^2^ While the question of risk stratification is often viewed from the perspective of identifying those at highest risk (safety driven), we now add a low-risk perspective: are there low-risk patients whom we can spare potentially costly and burdensome prolonged monitoring beyond the guideline-recommended minimum? Given the limitations of our work, our data are especially relevant for those at lowest risk of AF, and in those who are not able to commence monitoring immediately after symptom onset. Our data indicated that 14-day monitoring in low-risk stroke/TIA patients results in surprisingly low AF yields, while selecting for clinically relevant risk factors increases AF detection rates considerably. Due to the low overall AF yield and relatively low number of patients in our sample, we were unable to provide definitive answers to this question. Still, our data on AF yield in clinically relevant subgroups as well as our validation of risk models for post-stroke AF could be combined with that of other observational studies in order to increase our understanding of optimal screening strategies for AF detection after stroke or TIA. We emphasize, however, that it is ultimately up to physicians and other stakeholders in each particular care setting to decide whether the numbers needed to screen as reflected by our and previous studies are deemed sufficiently (cost-)efficient in their situation.

Our data underscore the need for a reliable triage test to identify patients in whom prolonged rhythm monitoring after TIA or stroke is associated with a fair chance of capturing AF. Given the low apparent yield in low-risk stroke/TIA patients, but with uncertainty around the optimal use of biomarkers as triage test for prolonged monitoring,^2^ further research could focus on strategies to use clinical parameters to select for prolonged monitoring. Depending on the available resources and expected burden to the patient of wearing extended ambulatory ECG monitoring, clinicians can use such work to decide on whether to extend monitoring duration in their particular patients.

### Strengths and limitations

A strength of our work is the prolonged Holter monitoring duration up to 14 days, which far exceeds the currently recommended continuous ECG recording duration for post-stroke patients. Moreover, there was a high rate of patient compliance with a large majority of patients wearing their Holter for 12 days or more. A further strength of our study was the relatively broad inclusion criteria compared to other work that focused ECG monitoring strategies on subgroups of patients, most notably patients with cryptogenic stroke or embolic stroke of undetermined source.^14,25,26,52^ This allowed for a closer validation of current guidelines whose recommendations for rhythm monitoring regard all ischemic stroke and TIA cases with undetermined origin.^1,2,8^ Another strength is our detailed documentation of reasons for exclusion to the study. This enabled us to demonstrate that almost a quarter of all stroke presentations arrived in our hospital with a known AF diagnosis at time of presentation, which potentially provided further context to the relatively low AF incidence during Holter monitoring. However, by carefully excluding patients with a history of AF or with de novo AF detected during comprehensive clinical and early outpatient clinical observation, our study allows for the assessment of truly new-onset AF. A final strength was the presentation of selected baseline characteristics among a random sample of non-included eligible patients which allowed a better assessment of the extent of sampling bias within our study.

The primary limitation of our study was sampling bias, resulting in a study population with lower risk of AF than the overall ischemic stroke/TIA population. The study’s logistic limitations as described above lowered the likelihood of severe stroke or tertiary care IAT patients to be included in our sample. To address this issue we presented limited baseline data from a random sample of non-included study-eligible patients in order to better understand the extent of sampling bias in our sample, which was considerable. Due to limitations imposed by the European Union’s GDPR we were unable to compare complete baseline characteristics of non-included eligible patients with those included in our study.^56^ The low AF incidence in our sample, as well as limitations to the scope of our dataset (e.g. incomplete data on MRI for stroke location particularly insular cortex, echocardiography for presence of patent foramen ovale, or biomarkers such as cardiac troponin) impaired our ability to assess the significance of risk factors and biomarkers associated with post-stroke AF detection.^34,45,57^ By still presenting baseline data stratified by AF detection during Holter monitoring, we aimed to contribute to potential future work on personalized monitoring approaches.^2^ While adherence to our 14-day study design was high among included patients, recent evidence points to the superiority implantable devices in detecting silent AF, which is reflected in the current ESO guideline’s recommendations.^2^ The use of 14-day Holter was thus a limitation in comparison to AF screening studies that employ implantable loop recorders, and potentially contributed to the low AF yield in our low-risk sample. The use of 2-lead Holter monitors in our study has been shown not to be associated with lower AF yield during post-stroke monitoring than 3- or 6-lead ambulatory monitors.^6^ Finally, our study does not contain follow-up for outcomes after AF detection, and is therefore unable to contribute to the work on stroke recurrence in post-stroke AF patients.^55^

## Conclusion

In conclusion, in low-risk patients with recent stroke or TIA, ESO guideline-recommended screening for AF resulted in a low AF yield, with limited additional value of monitoring up to 14 days. Our results underline the need for a personalized approach in determining a patient’s optimum duration for post-stroke non-invasive ambulatory monitoring.

## Supplemental Material

sj-docx-1-eso-10.1177_23969873221146027 – Supplemental material for 14-day Holter monitoring for atrial fibrillation after ischemic stroke: The yield of guideline-recommended monitoring duration Click here for additional data file. Supplemental material, sj-docx-1-eso-10.1177_23969873221146027 for 14-day Holter monitoring for atrial fibrillation after ischemic stroke: The yield of guideline-recommended monitoring duration by Jelle CL Himmelreich, Wim AM Lucassen, Jonathan M Coutinho, Ralf E Harskamp, Joris R de Groot and Henk CPM van Weert in European Stroke Journal
