# Supplementary material for: 14-day Holter monitoring for atrial fibrillation after ischemic stroke: The yield of guideline-recommended monitoring duration
Source: Eur Stroke J. 2022 Dec 23;8(1):157–67. doi: 10.1177/23969873221146027 (PMC10069211; doi:10.1177/23969873221146027)
Supplement: sj-docx-1-eso-10.1177_23969873221146027 – Supplemental material for 14-day Holter monitoring for atrial fibrillation after ischemic stroke: The yield of guideline-recommended monitoring duration [file sj-docx-1-eso-10.1177_23969873221146027.docx]

**Supplement to Himmelreich et al., “Fourteen-day Holter monitoring for atrial fibrillation after ischemic stroke: the yield of guideline-recommended monitoring duration”**

**Supplementary Table S1.** Comparison of selected baseline characteristics between the study sample (n = 379) and a 25% random sample of potentially eligible, non-included patients (n = 169).

|  | **Study sample (All, n=379)** | **Eligible**  **non-included (25% RS, n=169)** |
| --- | --- | --- |
| Female sex (%) | 42.2 | 49.7 |
| Age, years (Median [Q1-Q3]) | 63.0 (55.0-73.0) | 71.0 (60.0-80.0) |
| Qualifying event |  |  |
| Ischemic stroke (%) | 68.9 | 81.1 |
| TIA (%) | 31.1 | 18.9 |
| Stroke/TIA location |  |  |
| Middle cerebral artery (%) | 44.6 | 60.4 |
| Anterior cerebral artery (%) | 1.8 | 1.2 |
| Posterior cerebral artery (%) | 5.3 | 2.4 |
| Lacunar (%) | 12.4 | 13.0 |
| Retinal (%) | 4.2 | 3.0 |
| Vertebrobasilar (%) | 31.7 | 20.1 |
| Non-lacunar hemispheric stroke (%) | 34.6 | 56.8 |
| NIHSS at first presentation (Median [Q1-Q3]) | 1 (0-3) | 3 (1-9) |
| Intravenous thrombolysis (%) | 19.5 | 34.3 |
| Intra-arterial thrombectomy (%) | 7.1 | 17.2 |
| Heart failure (%) | 2.6 | 0 |
| Hypertension (%) | 46.4 | 45.6 |
| Diabetes (%) | 18.7 | 19.5 |
| Prior myocardial infarction (%) | 7.7 | 9.5 |
| Prior stroke/TIA/SE (%) | 21.9 | 31.4 |
| Vascular disease (%) | 12.9 | 21.9 |
| CHA_2_DS_2_-VASc (Median [Q1-Q3]) | 4 (3-5) | 5 (3-6) |
| Antiplatelet use (%) | 34.3 | 39.1 |
| ACE/ARB use (%) | 28.8 | 31.4 |
| Calcium antagonist use (%) | 18.5 | 17.8 |
| Diuretics use (%) | 13.2 | 21.3 |
| Statin use (%) | 36.9 | 38.5 |
| Insulin use (%) | 5.3 | 5.9 |
| Metformin use (%) | 12.9 | 14.8 |

ACE/ARB, angiotensin-converting enzyme inhibitor/angiotensin II receptor blocker; AF, atrial fibrillation; CHA_2_DS_2_-VASc, Congestive heart failure, Hypertension, Age ≥75 years (doubled), Diabetes mellitus, prior Stroke or TIA or thromboembolism (doubled), Vascular disease, Age 65 to 74 years, Sex category; NIHSS, National Institutes of Health Stroke Scale; Q1, 1^st^ quartile; Q3, 3^rd^ quartile; RS, random sample; SE, systemic embolism; TIA, transient ischemic attack.

**Supplementary Table S2.** Extended baseline characteristics of the total study sample (n = 379) stratified by AF presence on study Holter.

|  | **AF (n = 10)** | **No AF (n = 369)** |
| --- | --- | --- |
| Female sex (n,%) | 4 (40.0%) | 156 (42.3%) |
| Age, years (Median [Q1-Q3]) | 69.5 (63.8-71.8) | 63.0 (55.0-73.0) |
| Ethnicity |  |  |
| Caucasian/white (n,%) | 10 (100%) | 251 (68.0%) |
| African/black (n,%) | 0 (0%) | 71 (19.2%) |
| South Asian (n,%) | 0 (0%) | 19 (5.1%) |
| Asian (n,%) | 0 (0%) | 18 (4.9%) |
| Other (n,%) | 0 (0%) | 10 (3.3%) |
| Qualifying event |  |  |
| Ischemic stroke (n,%) | 9 (90.0%) | 252 (68.3%) |
| TIA (n,%) | 1 (10.0%) | 117 (31.7%) |
| Stroke/TIA location |  |  |
| Middle cerebral artery (n,%) | 8 (80.0%) | 161 (43.6%) |
| Anterior cerebral artery (n,%) | 0 (0%) | 7 (1.9%) |
| Posterior cerebral artery (n,%) | 1 (10.0%) | 19 (5.1%) |
| Lacunar (n,%) | 0 (0%) | 47 (12.7%) |
| Retinal (n,%) | 0 (0%) | 16 (4.3%) |
| Vertebrobasilar (n,%) | 1 (10.0%) | 119 (32.2%) |
| Non-lacunar hemispheric stroke (n,%) | 8 (80.0%) | 123 (33.3) |
| NIHSS at first presentation (Median [Q1-Q3]) | 7 (2-13) | 1 (0-3) |
| Ipsilateral carotid artery stenosis >50% (n,%) | 1 (10.0%) | 9 (2.4%) |
| Intravenous thrombolysis (n,%) | 5 (50.0%) | 69 (18.7%) |
| Intra-arterial thrombectomy (n,%) | 4 (40.0%) | 23 (6.2%) |
| Time to Holter, days (Median [Q1-Q3]) | 45 (8-60) | 35 (14-59) |
| Time to Holter ≤90 days (n,%) | 9 (90.0%) | 328 (88.9%) |
| Holter duration, days (Median [Q1-Q3]) | 14 (12-14) | 13 (12-14) |
| BMI, kg/m^2^ (Median [Q1-Q3]) | 25.3 (22.5-30.0) | 26.0 (24.0-29.1) |
| Missing (n,%) | 0 (0%) | 2 (0.5%) |
| SBP, mm Hg (Median [Q1-Q3]) | 156 (138-166) | 154 (135-172) |
| Missing (n,%) | 1 (10.0%) | 21 (5.7%) |
| DBP, mm Hg (Median [Q1-Q3]) | 78 (70-94) | 87 (77-99) |
| Missing (n,%) | 1 (10.0%) | 21 (5.7%) |
| Smoking |  |  |
| Current smoker (n,%) | 2 (20.0%) | 76 (20.6%) |
| Never smoked (n,%) | 2 (20.0%) | 134 (36.3%) |
| Former smoker (n,%) | 6 (60.0%) | 159 (43.1%) |
| Pack years, years (Median [Q1-Q3]) | 22 (4-45) | 7 (0-24) |
| Missing (n,%) | 0 (0%) | 8 (2.2%) |
| Alcohol units/day (Median [Q1-Q3]) | 2 (1-2) | 0 (0-1) |
| Family history of AF |  |  |
| Yes (n,%) | 3 (30.0%) | 45 (12.2%) |
| No (n,%) | 6 (60.0%) | 254 (68.8%) |
| Unknown (n,%) | 1 (10.0%) | 70 (19.0%) |
| Heart failure (n,%) | 0 (0%) | 10 (2.7%) |
| Hypertension (n,%) | 7 (70.0%) | 169 (45.8%) |
| Diabetes (n,%) | 0 (0%) | 71 (19.2%) |
| Prior myocardial infarction (n,%) | 1 (10.0%) | 28 (7.6%) |
| Prior stroke/TIA/SE (n,%) | 3 (30.0%) | 80 (21.7%) |
| Vascular disease (n,%) | 1 (10.0%) | 48 (13.0%) |
| Prior cardiac intervention (n,%) | 0 (0%) | 31 (8.4%) |
| Asthma or COPD (n,%) | 1 (10.0%) | 27 (7.3%) |
| Chronic kidney disease | 1 (10.0%) | 16 (4.3%) |
| CHA_2_DS_2_-VASc (Median [Q1-Q3]) | 4 (3-5) | 4 (3-5) |
| AS5F (Median [Q1-Q3]) | 65.9 (61.8-73.4) | 58.4 (51.6-65.2) |
| Re-CHARGE-AF (Median [Q1-Q3]) | 0.129 (0.095-0.168) | 0.112 (0.061-0.187) |
| Missing (n,%) | 1 (10.0%) | 23 (6.2%) |
| STAF (Median [Q1-Q3]) | 6 (5-6) | 5 (3-5) |
| Missing (n,%) | 0 (0%) | 22 (6.0%) |
| Antiplatelet use (n,%) | 4 (40.0%) | 126 (34.1%) |
| ACE/ARB use (n,%) | 4 (40.0%) | 105 (28.5%) |
| Calcium antagonist use (n,%) | 1 (10.0%) | 69 (18.7%) |
| Diuretics use (n,%) | 4 (40.0%) | 46 (12.5%) |
| Statin use (n,%) | 4 (40.0%) | 136 (36.9%) |
| Insulin use (n,%) | 0 (0%) | 20 (5.4%) |
| Metformin use (n,%) | 0 (0%) | 49 (13.3%) |
| eGFR, mL/min/1.73m^2^ (Median [Q1-Q3]) | 68 (55-81) | 77 (63-88) |
| Missing (n,%) | 0 (0%) | 7 (1.9%) |
| LDL cholesterol, mmol/L (Median [Q1-Q3]) | 2.32 (2.27-2.95) | 2.65 (1.93-3.42) |
| Missing (n,%) | 2 (20.0%) | 48 (13.0%) |
| Triglycerides, mmol/L (Median [Q1-Q3]) | 0.90 (0.78-1.32) | 1.36 (0.90-1.94) |
| Missing (n,%) | 2 (20.0%) | 48 (13.0%) |

ACE/ARB, angiotensin-converting enzyme inhibitor/angiotensin II receptor blocker; AF, atrial fibrillation; AS5F, Age, Stroke Severity NIHSS >5 to Find AF; BMI, body mass index; CHA_2_DS_2_-VASc, Congestive heart failure, Hypertension, Age ≥75 years (doubled), Diabetes mellitus, prior Stroke or TIA or thromboembolism (doubled), Vascular disease, Age 65 to 74 years, Sex category; COPD, chronic obstructive pulmonary disease; DBP, diastolic blood pressure; eGFR, estimated glomerular filtration rate; LDL, low-density lipoprotein; NIHSS, National Institutes of Health Stroke Scale; Q1, 1^st^ quartile; Q3, 3^rd^ quartile; Re-CHARGE-AF, Recalibrated Cohorts for Heart and Aging Research in Genomic Epidemiology for Atrial Fibrillation; SBP, systolic blood pressure; SE, systemic embolism; STAF, Score for the Targeting of Atrial Fibrillation; TIA, transient ischemic attack.

In case of missing data in each variable, number and percentage of missing are indicated.

**Supplementary Table S3.** Extended baseline characteristics of patients with over 48 hours of Holter recording and free of AF at 48 hours of monitoring (n = 362) stratified by AF presence on subsequent Holter.

|  | **AF (n = 3)** | **No AF (n = 359)** |
| --- | --- | --- |
| Female sex (n,%) | 1 (33.3%) | 152 (42.3%) |
| Age, years (Median [Q1-Q3]) | 70.0 (69.5-71.0) | 63.0 (55.0-73.0) |
| Ethnicity |  |  |
| Caucasian/white (n,%) | 3 (100%) | 245 (68.2%) |
| African/black (n,%) | 0 (0%) | 68 (19.1%) |
| South Asian (n,%) | 0 (0%) | 19 (5.3%) |
| Asian (n,%) | 0 (0%) | 18 (5.1%) |
| Other (n,%) | 0 (0%) | 9 (2.5%) |
| Qualifying event |  |  |
| Ischemic stroke (n,%) | 3 (100%) | 244 (68.0%) |
| TIA (n,%) | 0 (0%) | 115 (32.0%) |
| Stroke/TIA location |  |  |
| Middle cerebral artery (n,%) | 3 (100%) | 158 (44.0%) |
| Anterior cerebral artery (n,%) | 0 (0%) | 7 (1.9%) |
| Posterior cerebral artery (n,%) | 0 (0%) | 18 (5.1%) |
| Lacunar (n,%) | 0 (0%) | 44 (12.4%) |
| Retinal (n,%) | 0 (0%) | 16 (4.5%) |
| Vertebrobasilar (n,%) | 0 (0%) | 116 (32.6%) |
| Non-lacunar hemispheric stroke (n,%) | 3 (100%) | 120 (33.4%) |
| NIHSS at first presentation (Median [Q1-Q3]) | 18 (10-20) | 1 (0-3) |
| Ipsilateral carotid artery stenosis >50% (n,%) | 0 (0%) | 9 (2.5%) |
| Intravenous thrombolysis (n,%) | 2 (66.7%) | 67 (18.7%) |
| Intra-arterial thrombectomy (n,%) | 2 (66.7%) | 20 (5.6%) |
| Time to Holter, days (Median [Q1-Q3]) | 63 (32-70) | 36 (15-60) |
| Time to Holter ≤90 days (n,%) | 3 (100%) | 319 (88.9%) |
| Holter duration, days (Median [Q1-Q3]) | 14 (14-14) | 14 (12-14) |
| BMI, kg/m^2^ (Median [Q1-Q3]) | 21.9 (21.5-23.4) | 26.0 (24.0-29.1) |
| Missing (n,%) | 0 (0%) | 2 (0.6%) |
| SBP, mm Hg (Median [Q1-Q3]) | 148 (143-153) | 155 (136-171) |
| Missing (n,%) | 1 (33.3%) | 21 (5.8%) |
| DBP, mm Hg (Median [Q1-Q3]) | 70 (67-72) | 88 (77-99) |
| Missing (n,%) | 1 (33.3%) | 21 (5.8%) |
| Smoking |  |  |
| Current smoker (n,%) | 1 (33.3%) | 72 (20.1%) |
| Never smoked (n,%) | 1 (33.3%) | 131 (36.5%) |
| Former smoker (n,%) | 1 (33.3%) | 156 (43.5%) |
| Alcohol units/day (Median [Q1-Q3]) | 2 (1-2) | 0 (0-1) |
| Family history of AF |  |  |
| Yes (n,%) | 2 (66.7%) | 44 (12.3%) |
| No (n,%) | 1 (33.3%) | 248 (69.1%) |
| Unknown (n,%) | 0 (0%) | 67 (18.7%) |
| Heart failure (n,%) | 0 (0%) | 9 (2.5%) |
| Hypertension (n,%) | 2 (66.7%) | 168 (46.8%) |
| Diabetes (n,%) | 0 (0%) | 69 (19.2%) |
| Prior myocardial infarction (n,%) | 0 (0%) | 26 (7.2%) |
| Prior stroke/TIA/SE (n,%) | 0 (0%) | 79 (22.0%) |
| Vascular disease (n,%) | 0 (0%) | 46 (12.8%) |
| Prior cardiac intervention (n,%) | 0 (0%) | 29 (8.1%) |
| Asthma or COPD (n,%) | 1 (10.0%) | 25 (7.0%) |
| Chronic kidney disease | 1 (10.0%) | 16 (4.3%) |
| CHA_2_DS_2_-VASc (Median [Q1-Q3]) | 4 (4-5) | 4 (3-5) |
| AS5F (Median [Q1-Q3]) | 74.2 (67.8-75.0) | 58.4 (51.6-65.2) |
| Re-CHARGE-AF (Median [Q1-Q3]) | 0.109 (0.102-0.116) | 0.112 (0.062-0.186) |
| Missing (n,%) | 1 (33.3%) | 23 (6.4%) |
| STAF (Median [Q1-Q3]) | 6 (6-7) | 5 (3-5) |
| Missing (n,%) | 0 (0%) | 20 (5.6%) |
| Antiplatelet use (n,%) | 0 (0%) | 123 (34.3%) |
| ACE/ARB use (n,%) | 1 (33.3%) | 104 (29.0%) |
| Calcium antagonist use (n,%) | 0 (0%) | 68 (18.9%) |
| Diuretics use (n,%) | 2 (66.7%) | 46 (12.8%) |
| Statin use (n,%) | 2 (66.7%) | 133 (37.0%) |
| Insulin use (n,%) | 0 (0%) | 19 (5.3%) |
| Metformin use (n,%) | 0 (0%) | 48 (13.4%) |
| eGFR, mL/min/1.73m^2^ (Median [Q1-Q3]) | 52 (50-61) | 77 (63-88) |
| Missing (n,%) | 0 (0%) | 7 (1.9%) |
| LDL cholesterol, mmol/L (Median [Q1-Q3]) | 2.32 (2.31-2.32) | 2.65 (1.90-3.42) |
| Missing (n,%) | 1 (33.3%) | 46 (12.8%) |
| Triglycerides, mmol/L (Median [Q1-Q3]) | 0.77 (0.72-0.82) | 1.36 (0.90-1.94) |
| Missing (n,%) | 1 (33.3%) | 46 (12.8%) |

ACE/ARB, angiotensin-converting enzyme inhibitor/angiotensin II receptor blocker; AF, atrial fibrillation; AS5F, Age, Stroke Severity NIHSS >5 to Find AF; BMI, body mass index; CHA_2_DS_2_-VASc, Congestive heart failure, Hypertension, Age ≥75 years (doubled), Diabetes mellitus, prior Stroke or TIA or thromboembolism (doubled), Vascular disease, Age 65 to 74 years, Sex category; COPD, chronic obstructive pulmonary disease; DBP, diastolic blood pressure; eGFR, estimated glomerular filtration rate; LDL, low-density lipoprotein; NIHSS, National Institutes of Health Stroke Scale; Q1, 1^st^ quartile; Q3, 3^rd^ quartile; Re-CHARGE-AF, Recalibrated Cohorts for Heart and Aging Research in Genomic Epidemiology for Atrial Fibrillation; SBP, systolic blood pressure; SE, systemic embolism; STAF, Score for the Targeting of Atrial Fibrillation; TIA, transient ischemic attack.

In case of missing data in each variable, number and percentage of missing are indicated.

**Supplementary Figure S1.** Association between Holter initiation and day of first AF detection after qualifying event (n = 379)


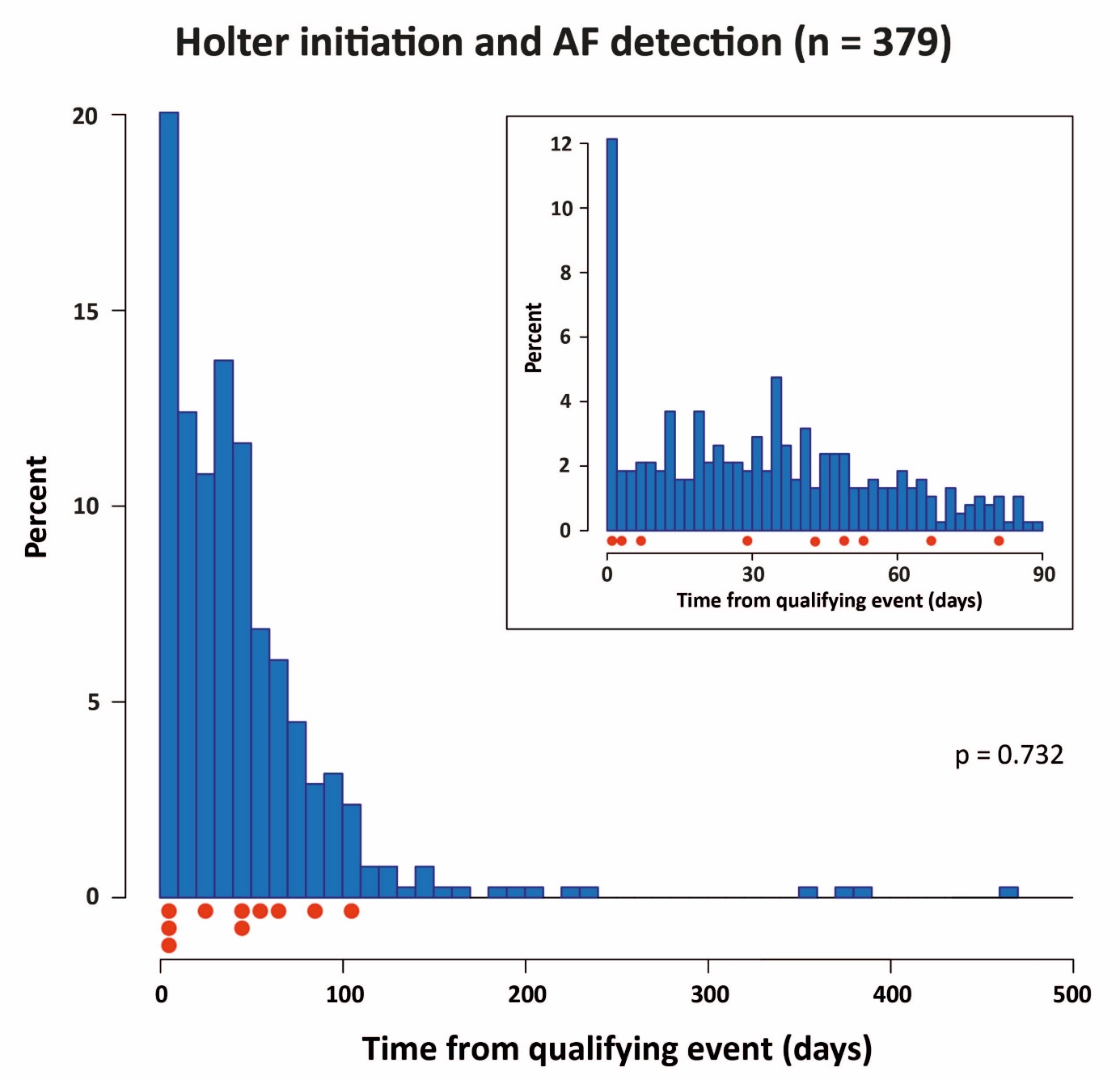


AF, atrial fibrillation.

Blue bars indicate percentage of included patients with Holter initiated after n days of the qualifying event. Red dots indicate the days from qualifying event at which each AF case was first detected (n = 10). P-value indicates the association between time from qualifying event to Holter and presence of AF on study Holter.
